# CO_2_ emissions in Latin America: a time series perspective based on fractional integration

**DOI:** 10.1007/s11356-023-29987-4

**Published:** 2023-09-30

**Authors:** Luis Rodrigo Asturias-Schaub, Luis Alberiko Gil-Alana

**Affiliations:** 1 Observatorio Económico Sostenible, Universidad del Valle, Guatemala, Guatemala; 2 NCID, DATAI, University of Navarra, Pamplona, Spain; 3 Universidad Francisco de Vitoria, Madrid, Spain; 4 Faculty of Economics and ICS, University of Navarra, 31080 Pamplona, Spain

**Keywords:** Emissions, Latin America, Persistence, Time trends, Fractional integration, B22, C01, C22, Q51, Q54

## Abstract

This article deals with the analysis of ${\mathrm{CO}}_{2}$ emissions in Latin America by using a long memory process based on fractional integration. Using data of ${\mathrm{CO}}_{2}$ emission and ${\mathrm{CO}}_{2}$ emissions per capita, for 32 Latin American and Caribbean countries, the results show significant differences according to the variable examined, the model used, and the country under examination. In particular, for the ${\mathrm{CO}}_{2}$ emissions, mean reversion is found in Belize and also under some circumstances in Antigua and Barbuda, Colombia, Dominica, Dominican Republic, Ecuador, Grenada, Honduras, Nicaragua, Panama, Peru, and Uruguay. Thus, shocks in these series have a transitory effect. With respect to the time trends, only for some Caribbean countries, namely, Antigua and Barbuda, Aruba, Bahamas, Cuba, and Jamaica, the trend is insignificant; on the other hand, large countries like Brazil, Mexico, and Argentina display the highest time trend coefficients; for the ${\mathrm{CO}}_{2}$ emissions per capita, there are eleven countries where mean reversion is detected, and there are ten that share a lack of significance for the trend. The most significant trends now take place in Trinidad and Tobago, British Virgin Islands, Barbados, and Guyana. Policy implications of the results obtained are reported at the end of the paper.

## Introduction

The Intergovernmental Panel on Climate Change —IPCC (Masson-Delmotte et al. 2019)— estimates that human activities have caused global warming of approximately 1.0 °C with respect to pre-industrial levels, with a probable range of 0.8 to 1.2 °C. Based on these measurements, global warming is likely to reach 1.5 °C between 2030 and 2052 if it continues to increase at the current rate. Moreover, carbon dioxide (${\mathrm{CO}}_{2}$) emissions have multiplied with the ensuing consequences. It is also true that this is not the only gas that contributes to global warming; there are other natural gases (methane, nitrous oxide) or artificial gases (fluorinated gases) that comprise the greenhouse gases (GHG). The sum and combination of gases cause climate change that is accompanied by climatic crises, and some regions in particular have been and will continue to be the most affected and vulnerable to the expected changes.

Carbon dioxide gases (${\mathrm{CO}}_{2}$) are generated from the combination of a chemical compound of two elements, carbon and oxygen, considering a ratio of one to two; hence, its molecular formula is ${\mathrm{CO}}_{2}$. This type of gas is present in the atmosphere in small amounts. ${\mathrm{CO}}_{2}$ plays a vital role in the Earth’s environment as it is considered one of the necessary ingredients for the life cycle of plants and animals.

Carbon is the fourth most abundant element in the universe, and on Earth, it is basic for life; the human being is 18% carbon. It is also essential when it mixes with oxygen and turns into carbon dioxide (${\mathrm{CO}}_{2}$): humans exhale it when they breathe, and plants need it for photosynthesis. The ${\mathrm{CO}}_{2}$ cycle has worked for thousands of years within a certain natural balance (Aunion and Palanelles 2019).

According to Metz et al. (2005), the main activities that cause the emission of ${\mathrm{CO}}_{2}$ include mainly anthropogenic activities such as the combustion of fossil fuels, coal, oil, and gas and the production of materials such as cement. There are also natural forms of ${\mathrm{CO}}_{2}$ emissions such as volcanic activity which is the main pathway to the surface for the transfer of carbon stored deep in the Earth. However, the problem arises when the circle of harmony is abruptly and continuously interrupted. The importance of analyzing the behavior and evolution of ${\mathrm{CO}}_{2}$ lies in the fact that its disproportionate emission represents around two-thirds of the greenhouse gases generated mainly as a result of the burning of fossil fuels (Metz et al. 2005).

Within the so-called planetary carbon cycle during the last 800,000 years, ${\mathrm{CO}}_{2}$ emissions have fluctuated between 170 and 330 parts per million, which have been considered acceptable levels for the sustainability of the planet. However, in the last 170 years, values have reached at least 415 parts per million (Sardá, 2021). Masson-Delmotte et al. (2019) detail that ${\mathrm{CO}}_{2}$ emissions in particular have increased by 40% since the pre-industrial era. It should be noted that gaseous carbon is absorbed through two pathways, the ocean and vegetation, and the emissions that cannot be absorbed remain in the atmosphere. As mentioned above, the largest ${\mathrm{CO}}_{2}$ emissions are mainly due to the burning of fossil fuels and deforestation.

The exponential growth rate in emissions has produced measurable changes in terms of changes in atmospheric, land, and sea temperatures. In particular, the oceans have absorbed around 30% of the anthropogenic carbon dioxide emitted, which has led to their acidification. In turn, the warming produces an increase in the level of the ocean by thermal expansion and due to the melting of the poles.

With regard to the exponential increase in the emissions of carbon dioxide, there are countries (Gil-Alana et al. 2016a, b) such as the BRICS (Brazil, Russia, India, China, and South Africa) whose trends are producing long-term effects on climate change. Likewise, the case of the G7 countries (US, UK, Japan, France, Italy, Germany, and Canada), whose ${\mathrm{CO}}_{2}$ emissions are directly related to their level of development, require an analysis focusing on the long-term dynamics and trends. The emissions of ${\mathrm{CO}}_{2}$ in the BRICS and G7 countries represent 70% of the total emissions of the world and three quarters of the accumulated carbon emissions.

Latin America and the Caribbean is one of the regions in the world most affected by Climate Change and external meteorological phenomena that are causing serious damage to health, life, food, water, energy, and socioeconomic development. When analyzing the period between 1998 and 2020, climate-related events and their impacts have claimed 312,000 lives and affected more than 277 million people in the region (World Meteorological Organization WMO 2021).

In particular, in the Latin American region, the year 2020 was one of the three warmest years on record in Mexico, Central America, and the Caribbean and the second warmest year in South America. Temperatures were 1 °C, 0.8 °C, and 0.6 °C above the 1981–2010 average, respectively (WMO 2021). The glaciers in the Chilean and Argentine Andes region have receded during the last decades. Ice mass loss has accelerated since 2010, in line with rising seasonal and annual temperatures, and there has been a significant reduction in annual precipitation in the region.

Furthermore, the intense drought in the southern Amazon and the Pantanal has been one of the most serious in the last 60 years, and 2020 was the year with the highest fire activity in the south of the Amazon. Similarly, throughout the Central American region, drought has been widespread, directly impacting crop yields and food production, aggravating food insecurity in many areas (WMO 2021).

At the end of 2020, hurricanes Eta and Iota reached category 4 intensity, particularly affecting Nicaragua, Honduras, and Guatemala. They made landfall in the same region in rapid succession, following identical trajectories that mainly affected rural areas with high rates of multidimensional poverty within the well-known “Central American Dry Corridor” (Bello and Peralta 2021).

In summary, the year 2020 witnessed a combination of extreme events in climatic terms. Heavy rains caused landslides, floods, hurricanes, droughts, warming oceans, and flash floods in rural and urban areas of Central America, the Caribbean, and South America. The effects of climate change are reflected in uncontrolled urbanization, the destruction of ecosystems that, together with factors such as poverty and malnutrition, generate a multidimensional problem (WMO 2021).

In view of the particular Latin American context in terms of vulnerability and impact, the importance of analyzing climate change by monitoring ${\mathrm{CO}}_{2}$ emissions over time and exploring the different effects generates an important topic to discuss in the development of the countries, considering that a significant percentage of the population in Latin America is living in highly vulnerable conditions.

Therefore, the principal objective of this research is to study the time patterns of ${\mathrm{CO}}_{2}$ emissions within Latin America and the Caribbean in order to detect the existence or not of persistence and trends, taking as a reference time series the period extending from 1960 to 2019, and considering that the data may present a long memory behavior, applying for this purpose fractional integration techniques. This methodology would appear to be very appropriate to determine the nature of the shocks, being transitory if the order of integration is smaller than 1 or permanent if this is equal to or higher than 1.

In recent years, Latin America has faced significant challenges in terms of CO_2_ emissions and their impact on the environment. The region’s growing industrialization, urbanization, and agricultural expansion have led to increased emissions, contributing to global concerns about climate change. Latin American countries vary in their contributions to CO_2_ emissions, with some being major emitters due to factors like energy production, deforestation, and transportation.

The impact of CO_2_ emissions in Latin America is multifaceted. One critical aspect is the role of deforestation, particularly in the Amazon rainforest, which serves as a significant carbon sink. The loss of forests not only releases stored carbon dioxide but also diminishes the forest’s ability to absorb CO_2_ from the atmosphere. This exacerbates the global carbon imbalance and accelerates climate change. Furthermore, the region’s vulnerability to climate change effects like extreme weather events, rising sea levels, and disrupted ecosystems poses a substantial risk to local communities and economies. Countries heavily reliant on agriculture, tourism, and natural resources can experience economic setbacks due to changing climate patterns. Vulnerable populations may face displacement and loss of livelihoods.

The Latin American region is remarkably heterogeneous in terms of climate, ecosystems, human population distribution, and cultural traditions. Land-use changes have become a major force driving ecosystem changes. Complex climatic patterns, which result in part from interactions of atmospheric flow with topography, intermingled with land-use and land-cover change, make it difficult to identify common patterns of vulnerability to climate change in the region. Water resources, ecosystems, agriculture and plantation forestry, sea-level rise, and human health may be considered the most important among the various sectors that may be impacted by climate change (Mccarthy et al. 2001).

The region is facing an asymmetrical dual challenge, since its contribution to total greenhouse gas emissions is limited, yet it is highly vulnerable to the effects of climate change. For example, agricultural activities are particularly sensitive to weather conditions and, thus, to climate change (Alatorre et al. 2014).

For its part, the IPCC (2022) report “Climate Change 2022: Impacts, Adaptation and Vulnerability” confirms that Central and South America are “highly exposed, vulnerable and strongly impacted by climate change,” a situation further aggravated by inequality, poverty, and increasing deforestation.

**Fig. 1:**
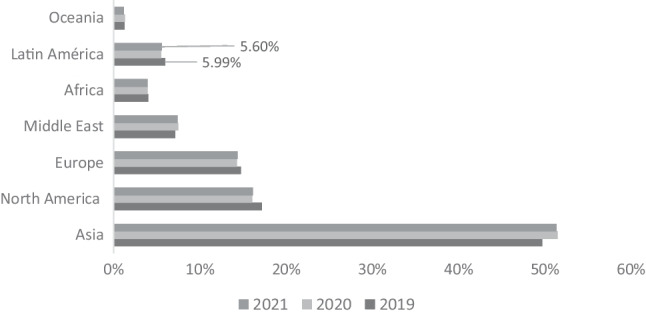
Percentage of ${\mathrm{CO}}_{2}$ emissions by region. Source: Global Carbon Atlas (2023)

Taking as reference the data of Global Carbon Budget (2023) in Fig. 1, we verify that less than 10% of ${\mathrm{CO}}_{2}$ emissions are generated in Latin America and the Caribbean. However, as mentioned above, it is one of the regions most impacted by climate change. Nevertheless, the cases of Brazil and Mexico are relevant as they are the most emitting countries in the region, ranking 12th and 13th worldwide respectively.

In Latin America and the Caribbean, accelerating climate change is increasing the frequency and intensity of extreme weather events and thus the economic impacts for the region. For example, the two category 4 hurricanes, Eta and Iota, affected more than 8 million people in Central America and caused damages estimated in the tens of billions of dollars. In Honduras, the average annual loss due to weather events is estimated at 2.3% of gross domestic product (World Bank 2021).

In ranking the impacts of extreme weather events between 2000 and 2019, five Caribbean countries ranked in the top 20 globally in terms of fatalities per capita, while in terms of economic losses as a share of GDP, eight of the top 20 countries are in the Caribbean (Eckstein et al. 2021).

Climate events reduce the income of the poorest 40% of the population by more than double the average population of Latin America and the Caribbean and could push between 2.4 and 5.8 million people in the region into extreme poverty by 2030 (Arga et al. 2020). Climate-related extreme events are also causing disruptions in energy systems. Disruptions in infrastructure services cost more than 1% of GDP on average across the region and up to 2% annually in several Central American countries (Hallegatte et al. 2019).

On average, in Latin America and the Caribbean, 56% of losses following weather events are due to disruptions in transportation services. Furthermore, the evolving effects of climate change are reducing productivity and adaptive capacity in many sectors. Climate change will have long-term negative impacts on yields of most crops in much of Latin America and the Caribbean, affecting food security and causing economic damage. For example, in Argentina, droughts could cause soybean yield losses of up to 50% by 2050 (Rozenberg et al. 2019).

Therefore, the application of ARFIMA models in the Latin American region creates interesting and innovative conclusions, since they have been applied in other geographical areas of the world with important results. For the Latin American region, it represents an important tool for discussion and analysis as it is one of the regions with more climate impacts particularly affecting the quality of life of developing countries. The methodology can create interesting contributions for regional public policy. In particular, the evidence of mean reversion obtained in six out of the 32 countries examined along with the 14 cases where the estimated order of integration is found to be smaller than 1 is something that may be related with the specific characteristic of the region of world under examination.

## Literature review

The importance of the analysis of greenhouse gas emissions and particularly ${\mathrm{CO}}_{2}$ emissions around vulnerability to climate change in Latin American countries has led to the need to model the historical behavior of the variable, considering the economic, environmental, and social impact that it generates.

The study of environmental variables and their relationship with econometrics is a field that has been increasingly applied. Chevallier (2009), Wang et al. (2013), Hammoudeh et al. (2014), and others have examined price drivers of ${\mathrm{CO}}_{2}$ emission allowance prices. Similarly, there are several papers that have examined the efficiency of carbon emission markets (Daskalakis 2008; Joyeux and Milunovich 2010; Charles et al. 2013, etc.).

By looking at the historical evaluation of the ${\mathrm{CO}}_{2}$ emissions, it is possible to determine the need of actions to recover the original levels after exogenous shocks in the data. Thus, if the series is stationary, the different shocks to its trend can be considered transitory. Thus, in the case of carbon dioxide, a policy intervention would not have to be considered urgent since the effects of the shocks on the variable may represent short-lived effects. In contrast, if the analysis of ${\mathrm{CO}}_{2}$ emissions confirms the non-stationary of the series, an effective articulation of a political nature is necessary, since by its non-stationary behavior, shocks and their effects will tend to present permanent effects.^1^^1^

The majority of the econometric methods investigating this variable and the effects of its shocks are related to stochastic convergence. Thus, for example, Strazicich and List (2003) examined ${\mathrm{CO}}_{2}$ emissions for 21 OECD countries within the years 1960–1997 by looking at the existence of unit roots in the log of the ratio of per capita emissions to average per capita emissions. The study posits that if per capita emissions converge stochastically over time, the effects and shocks of emissions would and should be temporary, considering that the data may be stationary. On the other hand, in the presence of a unit root, these clashes would be permanent and would suggest a lack of convergence. Strazicich and List (2003) claimed to find significant evidence for stochastic convergence of ${\mathrm{CO}}_{2}$ emissions per capita. In the same line, Im et al. (2003) applied conventional cross-sectional regressions and panel unit root tests to investigate conditional convergence in ${\mathrm{CO}}_{2}$ emissions, with their results supporting this hypothesis. Westerlund and Basher (2007) provided evidence of convergence in ${\mathrm{CO}}_{2}$ emissions in 16 industrialized countries and 12 developing countries using three panel unit root tests that control for cross-correlation through a factor model. On the other hand, Romero-Ávila (2008) examined the existence of stochastic and deterministic convergence of carbon dioxide (${\mathrm{CO}}_{2}$) emissions in 23 countries over the period 1960–2002. Their results support both stochastic and deterministic convergence in ${\mathrm{CO}}_{2}$ emissions, thus confirming the findings in Strazicich and List (2003). In both cases, this evidence supportive of convergence becomes apparent after controlling for multiple breaks, which account for shocks and policy interventions. Other authors such as Aldy (2006) applied seismic techniques in 23 OECD countries for the time period 1960–1999. They obtained ambiguous results and even when compared with a broader sample of countries, no evidence of convergence over time was found. Using a similar methodology, Panopoulou and Pantelidis (2009) concluded that in recent years, there may be two separate convergence clubs that converge at different equilibria. Barassi et al. (2007) employed a wide variety of tests, testing the null of a unit root and the null of stationarity for both individual series and for the OECD panel as a whole and allowing for cross-sectional dependencies within the panel. These authors found little evidence to suggest that per capita ${\mathrm{CO}}_{2}$ emissions within the OECD were converging. Christidou et al. (2013) investigated the stationarity of per capita ${\mathrm{CO}}_{2}$ emissions within 36 countries for the time period 1870–2006, where a robust avoidance of stationary in the series is mentioned. Finally, Heil and Selden (2010) used tests for unit roots in the analysis of 135 countries over the period 1950–1992, finding stationarity in only 20 of the 135 analyzed countries. In the results of Jiaxiong and Yunhui (2022) and Ericsson et al. (2022), the interpretation of structural breaks suggests that being able to identify a single break or multiple breaks would indicate that the climate may not only change gradually but may present changes abruptly.

In terms of long-term analysis, the evolution of ${\mathrm{CO}}_{2}$ emissions has presented moments of exponential growth related to population growth, industrialization, and historical moments in which certain countries, such as China and India, have undergone a process of productive transformation. These structural changes in the trend lines in ${\mathrm{CO}}_{2}$ emissions have been studied, for example, in the works of Altinay and Karagol (2004), Lee and Chang (2005, 2009), Lanne and Liski (2004); Mckitrick and Strazicich (2005), etc. In this context, allowing for structural breaks within the unit root hypothesis, Mckitrick and Strazicich (2005) examined 121 countries in the period 1950–2000 finding significant evidence against the unit root in most of the countries, together with significant evidence of structural breaks. Ordás Criado and Grether (2011) investigated the presence of stationarity in ${\mathrm{CO}}_{2}$ per capita in 166 countries during the period 1960–2012, concluding that convergence in the relative measure can be found in the presence of diverging and rising emissions in the unscaled data. Focusing on per capita emissions in levels, they highlight that strong divergence and increasing emissions are prevalent worldwide in the early period 1960–1980, but stabilization (in gaps and emissions) seems to take place after the oil price shocks of the 1970s.

In view of the above literature, it is clear that there is no consensus about the nature of ${\mathrm{CO}}_{2}$ emissions in terms of its stationary or non-stationary nature, and that the results vary substantially depending on the methodology used, the countries, and the time period examined. Nevertheless, most of these studies focus on unit root/stationarity methods that simply consider an integer degree of differentiation, i.e., 1 in case of non-stationary series (unit roots) or 0 in the presence of stationarity. The present paper goes one step further in the sense that we examine the ${\mathrm{CO}}_{2}$ emissions (and ${\mathrm{CO}}_{2}$ emissions per capita) from a fractional integration viewpoint, i.e., we allow for fractional degrees of differentiation. This literature belongs to the category of long memory models, so-named because observations which are far away in the past still have an influence on the present value, and a full description of these models is given in the following section. Using long memory models, we find the papers by Barassi et al. (2010) and Cuestas and Gil-Alana (2016), Claudio-Quiroga and Gil-Alana (2022), and Luis et al. (2023). In the latter, they examine the degree of persistence in the carbon emission allowance spot prices, using daily data for the period 2007–2014, and accounting for structural breaks and non-linearities in the data. The authors conclude that there is no evidence of non-linearities for the period examined. In a similar way, Luis et al. (2017) used fractional integration in a model that allows non-linear trends and structural breaks for ${\mathrm{CO}}_{2}$ emissions in the BRICS and G7 countries with a longer time series, including the last 150 years. In the paper, they showed that the results significantly change in both the degree of integration and the non-linearities depending on the countries under examination. Musolesi and Mazzanti (2014) studied non-linearities, heterogeneities, and unobserved effects in the carbon dioxide emissions-economic development relationship, while Gil-Alana et al. (2016a, b) examined the long memory property and the persistence of carbon emission allowance prices.

In view of the above literature, it is evident that there are no empirical papers studying the level of persistence in the ${\mathrm{CO}}_{2}$ emissions in Latin America with fractional integration techniques. In the following section, we precisely describe this methodology.

## Methodology

We start by providing some definitions of long memory processes. Using a time domain approach, a covariance stationary process, *x*(*t*), *t* = 0, ± 1, …, is said to be long memory if the infinite sum of the autocovariances is finite, i.e., defining the autocovariance function as *γ*(*u*) = *E*[(*x*(*t*) − *Ex*(*t*)) (*x*(*t* + *u*) – *Ex*(*t*))], *x*(*t*) displays the property of long memory if:

1 $${\sum }_{u=-\infty }^{u=\infty }\mid \gamma (u)\mid =\infty$$

Alternatively, a frequency domain characterization of long memory is the following. A process *x*(*t*) is long memory if its spectral density function, defined as the Fourier transform of the autocovariances, i.e.,

2 $$f(\lambda )=\frac{1}{2\pi }{\sum }_{u=-\infty }^{\infty }{\gamma }_{(u)}{e}^{i\lambda u}$$

displays a pole or singularity at any frequency on the spectrum, i.e.,

3 $$f\left(\lambda \right)\to \infty , \lambda \in \left[0,\pi \right)$$

In many cases, the singularity or pole in the spectrum occurs at the zero frequency, that is,

4 $$f\left(\lambda \right)\to \infty , as \lambda \to {0}^{+}$$

And in such a case, a very popular model to describe this behavior is the fractionally integrated model (or *I*(*d*) model) expressed as

5 $$(1-B{)}^{d}x(t)=u(t), t=\mathrm{1,2},...,$$

where *B* is the backshift operator (*Bx*(*t*) = *x*(*t* − 1)); *d* is a real value, and *u*(*t*) is an integrated of order 0 (and denoted by *I*(0)) process, indicating by this a covariance stationary process, where the infinite sum of the autocovariances is finite, or in the frequency domain, if the spectral density function is positive and bounded in the spectrum (i.e., 0 < *f*(*λ*) < *∞*, for all *λ*).

We are interested in the estimation of the time trend coefficients for the emissions in the context of long memory processes and, for this purpose, in the empirical application carried out in “Empirical results”, we consider the following model:

6 $$y\left(t\right)=\alpha +\beta t+x\left(t\right), (1-B{)}^{d}x(t)=u(t), t=\mathrm{1,2},...$$

where *y*(*t*) indicates the ${\mathrm{CO}}_{2}$ emissions (or emissions per capita) along time; *α* and *β* are unknown parameters referring respectively to an intercept and a linear time trend, and *x*(*t*) is assumed to be integrated of order *d*, or *I*(*d*), so that *u*(*t*) is *I*(0) or a short-memory process. Then, if *d* > 0, *x*(*t*) displays long memory, and the higher the value of *d* is, the higher the level of association between observations, noting that the polynomial in *B*, (1 – *B*)^*d*^ can be represented using a binomial expansion as

7 $$(1-B{)}^{d}={\sum }_{j=0}^{\infty }\left(\begin{array}{c}d\\ j\end{array}\right)(-1{)}^{j}{B}^{j}=1-dB+\frac{d(d-1)}{2}{B}^{2}-...,$$

which is valid for any real value *d*, and thus Eq. (5) can be expressed as:

8 $$x(t)=dx(t-1)-\frac{d(d-1)}{2}x(t-2)+...+u(t)$$

Thus, if *d* is a non-integer value, *x*(*t*) will be a function of all its past history, and the differencing parameter *d* can be taken as a measure of the degree of persistence in the data. On the other hand, depending on the value of *d*, different processes can be considered such as:

1. Anti-persistence, if *d* < 0
2. Short memory processes, if *d* = 0
3. Long memory covariance stationary, if 0 < *d* < 0.5
4. Long memory and mean reversion, if 0.5 ≤ *d* < 1
5. Unit roots or I(1) processes, if *d* = 1
6. Explosive processes, if *d* ≥ 1

We estimate *d* using a methodology presented in Robinson (1994) and that is based on a frequency domain representation of the Whittle function. It is a testing procedure that does not impose stationarity prior to the estimation; it has a standard limit distribution and is the most efficient method in the Pitman sense against local departures from the null.

## Data

Taking as reference the data obtained from the Global Carbon Budget (2021) for the period 1960–2019 with an annual frequency, Tables 1 and 2 analyze the descriptive results of each country of Latin America and the Caribbean, initially considering country emissions in terms of megatons of ${\mathrm{CO}}_{2}$ (${\mathrm{MtCO}}_{2})$ (in Table 1) and then emissions per capita in terms of tons of ${\mathrm{CO}}_{2}$ (Table 2).

**Table 1:** Descriptive statistics. ${\mathrm{CO}}_{2}$ emissions

Country	Mean	Std. Dev	Maximum	Minimum
Antigua and Barbuda	0.35	0.20	1.26	0.04
Argentina	122.76	43.55	192.37	48.76
Aruba	1.09	0.78	2.82	0.18
Bahamas	2.71	2.21	9.71	0.41
Barbados	0.88	0.41	1.61	0.17
Belize	0.30	0.17	0.64	0.04
Bolivia	8.26	6.23	22.58	1.00
Brazil	243.06	138.20	523.89	46.85
British Virgin Islands	0.09	0.07	0.21	0.004
Chile	41.94	23.54	85.83	13.48
Colombia	52.67	21.79	102.20	16.39
Costa Rica	4.05	2.63	8.51	0.49
Cuba	25.36	6.25	35.51	12.17
Dominica	0.08	0.06	0.18	0.01
Dominican Republic	11.99	8.00	27.38	1.03
Ecuador	19.04	12.79	43.21	1.56
El Salvador	3.67	2.19	6.85	0.58
Grenada	0.13	0.09	0.30	0.02
Guatemala	6.92	4.95	20.51	1.34
Guyana	1.56	0.43	2.39	0.62
Haiti	1.23	0.92	3.37	0.18
Honduras	4.09	3.24	10.93	0.62
Jamaica	7.26	2.52	11.58	1.47
Mexico	302.07	146.08	496.30	63.05
Nicaragua	2.80	1.46	5.55	0.53
Panama	4.89	3.23	12.50	1.00
Paraguay	2.80	2.15	8.27	0.30
Peru	27.38	13.18	57.15	8.17
Suriname	1.84	0.57	2.84	0.43
Trinidad and Tobago	21.85	14.16	46.96	1.31
Uruguay	5.45	1.19	8.59	3.17
Venezuela	115.10	45.76	192.75	51.88

**Table 2:** Descriptive statistics. ${\mathrm{CO}}_{2}$ emissions per capita

Country	Mean	Std. Dev	Maximum	Minimum
Antigua and Barbuda	4.93	3.11	19.78	0.68
Argentina	3.69	0.59	4.69	2.33
Aruba	13.50	6.88	27.87	2.87
Bahamas	12.38	12.41	49.26	4.60
Barbados	3.27	1.39	5.76	0.75
Belize	1.36	0.36	2.13	0.39
Bolivia	1.01	0.48	1.97	0.27
Brazil	1.53	0.51	2.58	2.58
British Virgin Islands	4.40	2.26	7.77	0.40
Chile	2.94	1.01	4.65	1.66
Colombia	1.55	0.24	2.09	1.02
Costa Rica	1.14	0.18	1.81	0.36
Cuba	2.49	0.50	3.46	1.67
Dominica	1.09	0.82	2.59	0.18
Dominican Republic	1.49	0.69	2.55	0.30
Ecuador	1.61	0.72	2.71	0.33
El Salvador	0.67	0.30	1.12	0.21
Grenada	1.26	0.84	2.81	0.16
Guatemala	0.63	0.22	1.17	0.31
Guyana	2.10	0.51	3.05	0.99
Haiti	0.15	0.07	0.30	0.02
Honduras	0.66	0.26	1.12	0.30
Jamaica	3.01	0.70	4.33	0.90
Mexico	3.42	0.91	4.45	1.59
Nicaragua	0.64	0.16	0.95	0.30
Panama	1.77	0.57	2.96	0.88
Paraguay	0.56	0.27	1.17	0.16
Peru	1.24	0.28	1.97	0.80
Suriname	4.29	1.15	6.68	1.45
Trinidad and Tobago	17.61	9.84	35.36	1.47
Uruguay	1.77	0.34	2.54	1.04
Venezuela	5.89	0.72	7.68	4.09

We see in Table 1 that the country that generates the most emissions in the region is Mexico with an average value of 302.07 ${\mathrm{MtCO}}_{2}$, followed by Brazil with 243.06 ${\mathrm{MtCO}}_{2}$, Argentina with 122.76, Venezuela with 115.10, and Colombia with an average emission of 52.67 ${\mathrm{MtCO}}_{2}$. There are countries where the average value of emissions is representative, for example, Chile, Peru, and Cuba with average values ranging between 41 and 25 ${\mathrm{MtCO}}_{2}$. On the other hand, the countries with the lowest emissions of ${\mathrm{MtCO}}_{2}$ are mainly concentrated in the Caribbean: Dominica (0.08 ${\mathrm{MtCO}}_{2})$, Virgin Islands (0.09), and Grenada (0.13)$.$ When analyzing the maximum values reached in ${\mathrm{MtCO}}_{2}$ emissions, once more countries such as Brazil (523.89), Mexico (496.30), Venezuela (192.75), and Argentina (192.37) display the highest values. These countries are at the same time the ones with the greatest variations in the emissions of ${\mathrm{MtCO}}_{2}$ in the region.

Table 2 displays the results of per capita emissions in tons of carbon dioxide ${\mathrm{TCO}}_{2};$ the countries with the highest emissions per person are found in the Caribbean islands: Trinidad and Tobago (17.61 ${\mathrm{TCO}}_{2}$), Aruba (13.50 ${\mathrm{TCO}}_{2}$), and Bahamas (12.38 ${\mathrm{TCO}}_{2}$), and fourth place is occupied by Venezuela (5.89 ${\mathrm{TCO}}_{2}$). The countries with the lowest ${\mathrm{CO}}_{2}$ emissions per capita in the region are Paraguay (0.56 ${\mathrm{TCO}}_{2}$), Guatemala (0.63 ${\mathrm{TCO}}_{2}$), Nicaragua (0.64 ${\mathrm{TCO}}_{2}$), and El Salvador (0.67 ${\mathrm{TCO}}_{2}$). The countries that present the maximum values in per capita emissions are the Bahamas, Trinidad and Tobago, Aruba, and Antigua and Barbuda, respectively. In terms of variations with respect to the mean, it is worth mentioning the important changes in the islands of Bahamas, Trinidad and Tobago, Aruba, and Antigua and Barbuda. It is noticeable throughout all the descriptive statistics of per capita emissions (in Table 2) that the countries in the Caribbean display the highest emissions and variations with respect to the mean.

## Empirical results

We separate this section in two sub-sections referring respectively to the ${\mathrm{CO}}_{2}$ emissions and the ${\mathrm{CO}}_{2}$ emissions per capita.

### CO_2_ emissions

Tables 3 and 5 display the estimates of the differencing parameter *d* in Eq. (6) under the assumption that *u*(*t*) is a white noise process in Table 3, and under autocorrelation in Table 5. In the two tables along with the estimates of *d*, we also report the 95% confidence bands of the non-rejection values of *d* using Robinson’s (1994) tests. The second column refers to the case where *α* and *β* in Eq. (6) are assumed to be zero a priori, that is, we do not consider deterministic terms in the model; the third column in the tables refers to the case with an intercept only, that is, imposing *β* = 0 a priori, while the last column corresponds to the model with a constant and a linear time trend, i.e., estimating *α* and *β* along with *d* from the data. We report in bold in the tables for each country the selected specification, based on the *t*-values on the *d*-differenced regressions, noting that the two equalities in Eq. (6) can be expressed as:

9 $$\widetilde{y}(t)=\alpha \widetilde{1}(t)+\beta \widetilde{t}(t)+u(t)$$

where

**Table 3:** Estimates of *d*. White noise errors

Country (CO_2_ emissions)	No terms	An intercept	An intercept and a time trend
Antigua and Barbuda	**0.77 (0.55, 1.10)**	0.76 (0.49, 1.10)	0.77 (0.54, 1.10)
Argentina	0.78 (0.58, 1.09)	1.01 (0.83, 1.32)	**0.99 (0.75, 1.31)**
Aruba	0.95 (0.79, 1.17)	**0.96 (0.81, 1.17)**	0.96 (0.81, 1.17)
Bahamas	**0.86 (0.73, 1.03)**	0.86 (0.72, 1.03)	0.86 (0.73, 1.03)
Barbados	0.79 (0.62, 1.05)	0.85 (0.73, 1.07)	**0.84 (0.67, 1.06)**
Belize	0.63 (0.55, 0.79)	0.68 (0.60, 0.80)	**0.47 (0.28, 0.73)**
Bolivia	0.94 (0.85, 1.07)	0.94 (0.85, 1.07)	**0.92 (0.81, 1.08)**
Brazil	1.02 (0.85, 1.31)	1.14 (0.96, 1.41)	**1.15 (0.92, 1.41)**
British Virgin Islands	1.09 (0.95, 1.29)	1.10 (0.97, 1.30)	**1.10 (0.95, 1.30)**
Chile	1.03 (0.89, 1.28)	1.12 (0.96, 1.43)	**1.13 (0.94, 1.45)**
Colombia	0.88 (0.73, 1.12)	0.76 (0.65, 0.93)	**0.76 (0.63, 0.95)**
Costa Rica	0.95 (0.82, 1.22)	0.96 (0.84, 1.22)	**0.95 (0.78, 1.23)**
Cuba	0.96 (0.81, 1.19)	**1.05 (0.88, 1.29)**	1.04 (0.88, 1.28)
Dominica	0.84 (0.76, 0.97)	0.87 (0.79, 1.00)	**0.83 (0.69, 0.99)**
Dominican Republic	0.97 (0.81, 1.33)	0.95 (0.80, 1.31)	**0.96 (0.75, 1.31)**
Ecuador	0.66 (0.59, 0.78)	0.71 (0.64, 0.82)	**0.48 (0.33, 0.70)**
El Salvador	0.96 (0.83, 1.16)	0.98 (0.87, 1.17)	**0.98 (0.85, 1.17)**
Grenada	0.79 (0.72, 0.89)	0.81 (0.75, 0.91)	**0.72 (0.62, 0.87)**
Guatemala	1.24 (1.11, 1.42)	1.22 (1.08, 1.40)	**1.24 (1.10, 1.42)**
Guyana	0.87 (0.73, 1.07)	0.78 (0.59, 1.00)	**0.81 (0.66, 1.01)**
Haiti	0.96 (0.83, 1.20)	0.98 (0.84, 1.22)	**0.97 (0.80, 1.24)**
Honduras	0.94 (0.86, 1.06)	0.94 (0.86, 1.05)	**0.92 (0.83, 1.06)**
Jamaica	0.96 (0.80, 1.16)	**0.95 (0.79, 1.15)**	0.95 (0.81, 1.14)
Mexico	0.83 (0.70, 1.05)	1.01 (0.89, 1.18)	**0.99 (0.85, 1.17)**
Nicaragua	0.83 (0.69, 1.10)	0.83 (0.71, 1.08)	**0.80 (0.63, 1.08)**
Panama	0.89 (0.80, 1.02)	0.88 (0.79, 1.01)	**0.86 (0.76, 1.01)**
Paraguay	**1.24 (1.06, 1.54)**	1.24 (1.06, 1.55)	1.26 (1.07, 1.57)
Peru	0.79 (0.66, 0.99)	0.78 (0.66, 0.97)	**0.76 (0.61, 0.97)**
Suriname	0.86 (0.67, 1.06)	0.83 (0.63, 1.05)	**0.85 (0.71, 1.05)**
Trinidad and Tobago	0.99 (0.86, 1.19)	1.01 (0.88, 1.20)	**1.01 (0.86, 1.20)**
Uruguay	0.83 (0.55, 1.08)	**0.76 (0.59, 1.03)**	0.76 (0.58, 1.03)
Venezuela	0.65 (0.50, 0.85)	**0.92 (0.77, 1.15)**	0.91 (0.72, 1.15)

The values in parenthesis refer to the 95% confidence band of the non-rejection values of *d* using Robinson’s (1994) tests. The values in bold refer to the selected specifications in relation with the deterministic terms

$\widetilde{y}(t)=(1-B{)}^{d}y(t), \widetilde{1}(t)=(1-B{)}^{d}1(t),$ and $\widetilde{t}(t)=(1-B{)}^{d}t(t)$, and based on the fact that *u*(*t*) in Eq. (6) is *I*(0) by construction, the *t*-values on the coefficients *α* and *β* apply. Thus, if both coefficients are statistically significant, we keep that model as the appropriate one; however, if *β* is found to be insignificant, we move to the model in column 3 with an intercept; finally, if both coefficients are statistically insignificant, we choose the model with no deterministic terms.

Table 3 reports the estimates of *d* for each series under the assumption that the error term is a white noise process. Thus, the whole structure of dependence is captured throughout the differencing parameter *d*. As earlier mentioned, we report the values of *d* under three different scenarios:

1. Imposing a priori that *α* = *β* = 0 in Eq. (6), i.e., we do not consider any deterministic term in the model
2. Imposing that only *β* = 0 a priori in Eq. (6), i.e., including an intercept
3. Allowing *α* and *β* being freely estimated from the data along with the other parameters, i.e., we permit here a linear time trend in the model

Table 4 reports the selected specifications for each series. We notice evidence of mean reversion (statistical significance of *d* < 1) for Ecuador, Belize, Grenada, Colombia, Peru, and Dominica. Thus, shocks are transitory in these countries. For the majority of cases, however, we cannot reject *d* = 1, and for Guatemala and Paraguay, the estimate of the differencing parameter *d* is even significantly higher than 1. Thus, except for the abovementioned six countries, shocks in the emissions are expected to be permanent requiring policy actions if we would like the series to return to their original long-term projections. The time trends are significant (and positive) in 24 out of the 32 countries examined. The highest coefficients correspond to Brazil (6.707), Mexico (6.398), Argentina (2.213), Chile (1.170), and Colombia (1.356), which is clearly related with the economic weight of these countries. According to the Economic Commission for Latin America and the Caribbean, ECLAC (2022), the mentioned countries represent 80% of the Latin American GDP, implying a clear relationship between the number of emissions and the economic activity in these countries. No significant trends are found in Antigua and Barbuda, Aruba, Bahamas, Cuba, Jamaica, Paraguay, Uruguay, or Venezuela.

**Table 4:** Estimated coefficients in the selected models in Table 3

Country (CO_2_ emissions)	No terms	An intercept	An intercept and a time trend
Antigua and Barbuda	0.77 (0.55, 1.10)	–-	–-
Argentina	0.99 (0.75, 1.31)	46.558 (7.97)	2.213 (3.04)
Aruba	0.96 (0.81, 1.17)	0.621 (1.95)	–-
Bahamas	0.86 (0.73, 1.03)	–-	–-
Barbados	0.84 (0.67, 1.06)	0.158 (1.81)	0.018 (2.88)
Belize	0.47 (0.28, 0.73)^a^	0.021 (0.83)	0.0096 (12.15)
Bolivia	0.92 (0.81, 1.08)	0.559 (1.67)	0.362 (4.51)
Brazil	1.15 (0.92, 1.41)	41.100 (3.19)	6.707 (2.30)
British Virgin Islands	1.10 (0.95, 1.30)	0.0016 (0.20)	0.0026 (1.76)
Chile	1.13 (0.94, 1.45)	12.352 (4.56)	1.170 (2.06)
Colombia	0.76 (0.63, 0.95)^a^	14.934 (4.17)	1.356 (6.64)
Costa Rica	0.95 (0.78, 1.23)	0.341 (1.71)	0.136 (4.35)
Cuba	1.05 (0.88, 1.29)	13.671 (6.55)	–-
Dominica	0.83 (0.69, 0.99)^a^	0.0066 (0.76)	0.0027 (4.58)
Dominican Republic	0.96 (0.75, 1.31)	0.555 (0.58)	0.444 (4.19)
Ecuador	0.48 (0.33, 0.70)^a^	− 1.111 (− 1.60)	0.695 (12.03)
El Salvador	0.98 (0.85, 1.17)	0.522 (1.76)	0.095 (2.68)
Grenada	0.72 (0.62, 0.87)^a^	0.0105 (1.90)	0.0046 (7.84)
Guatemala	1.24 (1.10, 1.42)^b^	1.113 (2.12)	0.336 (2.02)
Guyana	0.81 (0.66, 1.01)	0.657 (3.82)	0.027 (2.35)
Haiti	0.97 (0.80, 1.24)	0.232 (1.40)	0.051 (2.55)
Honduras	0.92 (0.83, 1.06)	0.402 (1.11)	0.173 (4.99)
Jamaica	0.95 (0.79, 1.15)	1.551 (1.90)	–-
Mexico	0.99 (0.85, 1.17)	56.600 (3.77)	6.398 (3.42)
Nicaragua	0.80 (0.63, 1.08)	0.436 (1.79)	0.083 (5.32)
Panama	0.86 (0.76, 1.01)	0.741 (1.42)	0.189 (4.61)
Paraguay	1.24 (1.06, 1.54)^b^	–-	–-
Peru	0.76 (0.61, 0.97)^a^	7.296 (2.30)	0.757 (4.18)
Suriname	0.85 (0.71, 1.05)	0.423 (1.95)	0.035 (2.15)
Trinidad and Tobago	1.01 (0.86, 1.20)	1.975 (0.87)	0.595 (1.97)
Uruguay	0.76 (0.59, 1.03)	4.443 (7.19)	–-
Venezuela	0.92 (0.77, 1.15)	57.428 (4.88)	–-

The values in parenthesis in columns 3 and 4 are the *t*-values associated to the estimated coefficients

^a^Mean evidence of “mean reversion” at the 95% level

^b^Estimates of *d* which are significantly higher than 1

In Tables 5 and 6, we allow for autocorrelation in the error term, i.e., *u*(*t*) in Eq. (6) is time dependent. However, instead of imposing a classical autoregressive moving average (ARMA) process for the error term, we use here an old non-parametric methodology (Bloomfield 1973) that approximates this type of structures with a lower number of parameters, and which the spectral density function (in log) is similar to the one produced by an AR model. In addition, this Bloomfield (1973) approximation accommodates very well in the context of the tests of Robinson (1994) used in this article (see, e.g., Gil-Alana 2004). Table 5 displays the results for the three abovementioned cases in relation with the deterministic terms (no terms; with an intercept, and with an intercept and a linear time trend), while in Table 6, we report the specific coefficients of the selected models.

**Table 5:** Estimates of *d* under the assumption of autocorrelated errors

Country (CO_2_ emissions)	No terms	An intercept	An intercept and a time trend
Antigua and Barbuda	**0.20 (− 0.39, 0.76)**	0.06 (− 0.36, 0.75)	0.06 (− 0.29, 0.75)
Argentina	0.56 (0.44, 0.92)	0.77 (0.60, 1.18)	**0.54 (0.09, 1.16)**
Aruba	0.90 (0.48, 1.38)	**0.93 (0.60, 1.39)**	0.93 (0.51, 1.39)
Bahamas	**1.31 (0.90, 1.91)**	1.31 (0.88, 1.91)	1.32 (0.89, 1.97)
Barbados	0.75 (0.49, 1.49)	0.94 (0.67, 1.51)	**0.92 (0.36, 1.53)**
Belize	0.64 (0.51, 0.90)	0.68 (0.54, 0.86)	**0.29 (− 0.14, 0.82)**
Bolivia	1.16 (0.94, 1.54)	1.13 (0.92, 1.55)	**1.17 (0.90, 1.60)**
Brazil	0.81 (0.66, 1.23)	0.97 (0.77, 1.72)	**0.80 (0.30, 1.78)**
British Virgin Islands	**1.22 (0.94, 1.64)**	1.25 (0.97, 1.66)	1.28 (0.89, 1.65)
Chile	0.88 (0.70, 1.20)	0.83 (0.67, 1.09)	**0.81 (0.56, 1.10)**
Colombia	1.30 (0.99, 1.62)	0.54 (0.23, 1.48)	**1.05 (0.57, 1.63)**
Costa Rica	0.79 (0.64, 1.05)	0.81 (0.65, 1.05)	**0.68 (0.40, 1.05)**
Cuba	0.94 (0.65, 1.30)	**0.95 (0.61, 1.37)**	0.96 (0.67, 1.38)
Dominica	**1.08 (0.87, 1.91)**	1.09 (0.87, 1.84)	1.20 (0.81, 1.82)
Dominican Republic	0.69 (0.55, 0.92)	0.70 (0.56, 0.90)	**0.42 (0.10, 0.89)**
Ecuador	0.73 (0.53, 1.06)	0.77 (0.58, 1.10)	**0.46 (− 0.02, 1.14)**
El Salvador	1.10 (0.81, 1.53)	1.06 (0.81, 1.44)	**1.05 (0.76, 1.45)**
Grenada	0.97 (0.84, 1.17)	1.03 (0.89, 1.24)	**1.03 (0.80, 1.29)**
Guatemala	1.33 (0.85, 1.93)	**1.43 (0.77, 1.84)**	1.42 (0.76, 1.83)
Guyana	1.06 (0.67, 1.45)	0.71 (0.06, 1.38)	**0.88 (0.20, 1.36)**
Haiti	0.86 (0.67, 1.15)	0.85 (0.64, 1.14)	**0.80 (0.56, 1.18)**
Honduras	1.09 (0.93, 1.31)	1.10 (0.96, 1.31)	**1.13 (0.93, 1.36)**
Jamaica	1.13 (0.69, 1.63)	**1.15 (0.68, 1.64)**	1.13 (0.76, 1.59)
Mexico	0.89 (0.67, 1.35)	**1.33 (0.87, 1.69)**	1.33 (0.87, 1.68)
Nicaragua	0.62 (0.46, 0.85)	0.66 (0.50, 0.84)	**0.48 (0.19, 0.82)**
Panama	1.09 (0.91, 1.35)	1.07 (0.88, 1.31)	**1.09 (0.88, 1.34)**
Paraguay	0.90 (0.66, 1.25)	0.90 (0.61, 1.22)	**0.89 (0.53, 1.26)**
Peru	0.83 (0.62, 1.17)	0.78 (0.56, 1.11)	**0.75 (0.49, 1.11)**
Suriname	1.04 (0.63, 1.55)	**0.97 (0.14, 1.60)**	1.00 (0.63, 1.55)
Trinidad and Tobago	**1.06 (0.75, 1.52)**	1.17 (0.84, 1.58)	1.15 (0.63, 1.61)
Uruguay	0.62 (0.22, 1.03)	0.44 (0.14, 0.85)	**0.39 (0.07, 0.84)**
Venezuela	0.48 (0.38, 0.77)	0.87 (0.33, 1.37)	**0.87 (0.41, 1.34)**

The values in parenthesis refer to the 95% confidence band of the non-rejection values of *d* using Robinson’s (1994) tests. The values in bold refer to the selected specifications in relation with the deterministic terms

**Table 6:** Estimated coefficients in the selected models in Table 5

Country (CO_2_ emissions)	No terms	An intercept	An intercept and a time trend
Antigua and Barbuda	0.20 (− 0.39, 0.76)^a^	–-	–-
Argentina	0.54 (0.09, 1.16)	47.414 (7.37)	2.406 (11.02)
Aruba	0.93 (0.60, 1.39)	0.624 (1.97)	–-
Bahamas	1.31 (0.90, 1.91)	–-	–-
Barbados	0.92 (0.36, 1.53)	0.156 (1.76)	0.017 (2.08)
Belize	0.29 (− 0.14, 0.82)^a^	0.015 (0.44)	0.0096 (9.77)
Bolivia	1.17 (0.90, 1.60)	0.733 (1.92)	0.359 (1.86)
Brazil	0.80 (0.30, 1.78)	36.183 (2.73)	7.358 (8.63)
British Virgin Islands	1.22 (0.94, 1.64)	–-	–-
Chile	0.81 (0.56, 1.10)	11.854 (4.47)	1.210 (6.85)
Colombia	1.05 (0.57, 1.63)	14.921 (4.05)	1.479 (2.58)
Costa Rica	0.68 (0.40, 1.05)	0.197 (0.63)	0.138 (9.67)
Cuba	0.95 (0.61, 1.37)	13.728 (6.60)	–-
Dominica	1.08 (0.87, 1.91)	–-	–-
Dominican Republic	0.42 (0.10, 0.89)^a^	− 0.769 (− 0.56)	0.436 (10.83)
Ecuador	0.46 (− 0.02, 1.14)	− 1.228 (− 0.47)	0.696 (8.78)
El Salvador	1.05 (0.76, 1.45)	0.529 (1.78)	0.093 (2.02)
Grenada	1.03 (0.80, 1.29)	0.017 (1.50)	0.0044 (2.61)
Guatemala	1.43 (0.77, 1.84)	1.305 (2.78)	–-
Guyana	0.88 (0.20, 1.36)	0.644 (3.68)	0.027 (1.88)
Haiti	0.80 (0.56, 1.18)	0.186 (1.13)	0.050 (4.71)
Honduras	1.13 (0.93, 1.36)	0.472 (1.88)	0.173 (2.41)
Jamaica	1.15 (0.68, 1.64)	1.337 (1.66)	–-
Mexico	1.33 (0.87, 1.69)	61.583 (4.46)	–-
Nicaragua	0.48 (0.19, 0.82)^a^	0.401 (1.65)	0.082 (9.46)
Panama	1.09 (0.88, 1.34)	0.821 (1.81)	0.197 (2.14)
Paraguay	0.89 (0.53, 1.26)	0.127 (0.50)	0.131 (5.99)
Peru	0.75 (0.49, 1.11)	7.289 (2.24)	0.755 (4.20)
Suriname	0.97 (0.14, 1.60)	0.432 (1.98)	–-
Trinidad and Tobago	1.06 (0.75, 1.52)	–-	–-
Uruguay	0.39 (0.07, 0.84)^a^	4.383 (10.23)	0.036 (2.93)
Venezuela	0.87 (0.41, 1.34)	54.637 (4.71)	1.462 (1.95)

The values in parenthesis in columns 3 and 4 are the *t*-values associated to the estimated coefficients

^a^Mean evidence of mean reversion at the 95% level

The first noticeable feature observed in Tables 5 and 6 is that evidence of mean reversion is found in the cases of Antigua and Barbuda, Belize, Uruguay, Dominican Republic, and Nicaragua. For the rest of the countries, we cannot reject the unit root null hypothesis (*d* = 1). Thus, Belize is the only country that displays mean reversion under the two types of specifications for the error term. However, the most significant time trends take place at Brazil (7.358), Argentina (2.406), Colombia (1.479), Chile (1.210), and Venezuela (1.462) (surprisingly, Mexico displays now an insignificant trend), and they are insignificant also for Antigua and Barbuda, Aruba, Bahama, British Virgin Islands, Cuba, Dominica, Guatemala, Jamaica, Surinam, and Trinidad and Tobago. Summarizing then the results in terms of the time trends, we see that Brazil, Argentina, Chile, and Colombia, along with Mexico (under the assumption of no autocorrelation) and Venezuela (with autocorrelated errors), are the countries that display significant positive trends. This is clearly related with their economic activity and geographical characteristics, and it is possible to relate them to the initiatives of the NGO Climate Analytics and New Climate Institute (2022), which analyzes the commitments and actions of governments and compares them against the objectives of the Paris Agreement. These results are presented in the Climate Action Tracker (CAT) where the countries with the highest greenhouse gas emissions are reflected, since they represent 80% of total emissions in the region. These major emitters include the six largest economies in Latin America: Brazil, Mexico, Argentina, Colombia, Chile, and Peru. According to the Climate Policy Update tracking CAT, the initiatives of the first four countries are rated as “highly insufficient.” While in the case of Chile and Peru, they are ahead, although they are still considered “insufficient” emphasizing that nine out of the twenty most vulnerable countries in the world to climate change are in the Latin American region, which supports the hypothesis of a highly representative trend in the emissions.

### CO_2_ emissions per capita

Table 7 displays the estimates of *d* under the assumption of white noise errors. We notice here that the time trend is required in 19 countries; for another group of 11 countries, the intercept is sufficient to describe the deterministic part, and only Antigua and Barbuda and Bahamas display no deterministic terms in their models. The highest time trend coefficients occur now in the cases of Trinidad and Tobago (0.4248) and British Virgin Islands (0.0972) followed by Barbados (0.0619) and Grenada (0.0413) (see Table 8), which are all small island states in the Caribbean. In the particular case of Trinidad and Tobago, the composition of its energy matrix stands out, since it obtains practically all of its energy from hydrocarbons. Therefore, renewable energies play a marginal role (Latin American Energy Organization —LAEO; International Energy Agency 2021). Trinidad and Tobago’s economic structure is highly dependent on oil and gas exploitation and generation. In 2010, Trinidad and Tobago was the world’s second largest producer of greenhouse gases per capita (Oxford Business Group 2021). By 2018, the country’s per capita CO_2_ emissions related to fuel combustion continued to outpace other countries in Latin America and the Caribbean by a wide margin (International Energy Agency 2022). In the case of Barbados, 93% of its electricity is generated by fossil fuels while the remaining percentage is generated by solar energy (LAEO 2021). One of the main reasons for the high per capita ${\mathrm{CO}}_{2}$ emissions in this country is related to the fact that 18.93% of the population is employed in the industrial sector, which includes mining, quarrying, manufacturing, construction, electricity, gas, and water (Statista 2022). The island of Barbados is greatly affected by various aspects of climate change, including rising sea levels and increased storm intensity. As such, the shift towards renewable energy on the island is an attempt to mitigate some of the effects of climate change that the country is undergoing (Illes 2021). The island is also experiencing a significant increase in the amount of energy that is being generated from renewable energy.

**Table 7:** Estimates of *d* under the assumption of white noise errors

Country: CO_2_ per capita	No terms	An intercept	An intercept and a time trend
Antigua and Barbuda	**0.77 (0.56, 1.10)**	0.75 (0.49, 1.10)	0.76 (0.51, 1.10)
Argentina	0.89 (0.72, 1.13)	**1.03 (0.83, 1.32)**	1.03 (0.83, 1.32)
Aruba	0.79 (0.62, 1.02)	**0.80 (0.65, 1.01)**	0.80 (0.65, 1.01)
Bahamas	**0.88 (0.75, 1.05)**	0.88 (0.75, 1.04)	0.88 (0.75, 1.04)
Barbados	0.78 (0.59, 1.04)	0.84 (0.71, 1.06)	**0.84 (0.68, 1.06)**
Belize	0.70 (0.50, 0.91)	0.63 (0.50, 0.85)	**0.68 (0.54, 0.87)**
Bolivia	0.84 (0.71, 1.03)	0.84 (0.72, 1.03)	**0.82 (0.67, 1.03)**
Brazil	0.94 (0.71, 1.25)	**1.20 (0.98, 1.47)**	1.19 (0.97, 1.47)
British Virgin Islands	0.87 (0.72, 1.08)	0.91 (0.79, 1.10)	**0.90 (0.75, 1.10)**
Chile	0.99 (0.82, 0.25)	**1.15 (0.94, 1.51)**	1.15 (0.94, 1.51)
Colombia	1.02 (0.86, 1.26)	0.79 (0.61, 1.02)	**0.83 (0.70, 1.02)**
Costa Rica	0.94 (0.70, 1.25)	0.97 (0.75, 1.29)	**0.97 (0.75, 1.28)**
Cuba	0.93 (0.76, 1.14)	**1.03 (0.87, 1.27)**	1.03 (0.87, 1.27)
Dominica	0.84 (0.76, 0.97)	0.88 (0.79, 0.99)	**0.83 (0.71, 0.99)**
Dominican Republic	0.97 (0.70, 1.32)	0.95 (0.74, 1.30)	**0.96 (0.74, 1.29)**
Ecuador	0.59 (0.46, 0.80)	0.68 (0.58, 0.85)	**0.64 (0.50, 0.84)**
El Salvador	0.90 (0.75, 1.12)	0.94 (0.81, 1.14)	**0.94 (0.80, 1.13)**
Grenada	0.76 (0.69, 0.87)	0.80 (0.74, 0.91)	**0.71 (0.59, 0.88)**
Guatemala	0.86 (0.71, 1.06)	0.75 (0.58, 0.97)	**0.77 (0.62, 0.98)**
Guyana	1.08 (0.92, 1.28)	1.05 (0.86, 1.27)	**1.05 (0.87, 1.28)**
Haiti	0.82 (0.66, 1.08)	0.86 (0.70, 1.12)	**0.83 (0.63, 1.12)**
Honduras	0.83 (0.70, 1.07)	0.80 (0.71, 0.96)	**0.77 (0.65, 0.96)**
Jamaica	0.98 (0.83, 1.16)	**0.95 (0.79, 1.14)**	0.95 (0.81, 1.13)
Mexico	0.85 (0.68, 1.06)	**1.02 (0.90, 1.18)**	1.02 (0.90, 1.18)
Nicaragua	0.89 (0.72, 1.14)	0.79 (0.57, 1.10)	**0.82 (0.64, 1.10)**
Panama	0.87 (0.73, 1.05)	0.80 (0.67, 0.97)	**0.80 (0.67, 0.98)**
Paraguay	1.04 (0.86, 1.29)	1.03 (0.82, 1.31)	**1.03 (0.82, 1.31)**
Peru	0.91 (0.77, 1.10)	**0.82 (0.67, 1.04)**	0.83 (0.69, 1.04)
Suriname	0.91 (0.79, 1.09)	**0.89 (0.74, 1.07)**	0.90 (0.77, 1.07)
Trinidad and Tobago	0.90 (0.75, 1.11)	0.92 (0.79 1.11)	**0.91 (0.76 1.11)**
Uruguay	0.86 (0.70, 1.11)	**0.79 (0.61, 1.06)**	0.79 (0.61, 1.06)
Venezuela	0.70 (0.51, 0.95)	**0.70 (0.41, 1.02)**	0.75 (0.55, 1.03)

The values in parenthesis refer to the 95% confidence band of the non-rejection values of *d* using Robinson’s (1994) tests. The values in bold refer to the selected specifications in relation with the deterministic terms

**Table 8:** Estimated coefficients in the selected models in Table 7

Country: CO_2_ per capita	No terms	An intercept	An intercept and a time trend
Antigua and Barbuda	0.77 (0.56, 1.10)	–-	–-
Argentina	1.03 (0.83, 1.32)	2.3742 (13.96)	–-
Aruba	0.80 (0.65, 1.01)	11.5298 (3.06)	–-
Bahamas	0.88 (0.75, 1.05)	–-	–-
Barbados	0.84 (0.68, 1.06)	0.7137 (2.16)	0.0619 (2.56)
Belize	0.68 (0.54, 0.87)^a^	0.5369 (3.60)	0.0190 (2.79)
Bolivia	0.82 (0.67, 1.03)	0.2397 (2.63)	0.0281 (4.48)
Brazil	1.20 (0.98, 1.47)	0.6395 (8.81)	–-
British Virgin Islands	0.90 (0.75, 1.10)	0.3629 (0.92)	0.0972 (2.68)
Chile	1.15 (0.94, 1.51)	1.6277 (8.84)	–-
Colombia	0.83 (0.70, 1.02)	1.0260 (11.55)	0.0152 (2.19)
Costa Rica	0.97 (0.75, 1.28)	0.3465 (3.80)	0.0223 (2.12)
Cuba	1.03 (0.87, 1.27)	1.9198 (9.51)	–-
Dominica	0.83 (0.71, 0.99)^a^	0.1227 (1.00)	0.0373 (4.29)
Dominican Republic	0.96 (0.74, 1.29)	0.2766 (2.22)	0.0377 (2.72)
Ecuador	0.64 (0.50, 0.84)^a^	0.3462 (1.66)	0.0364 (4.24)
El Salvador	0.94 (0.80, 1.13)	0.2116 (3.74)	0.0127 (2.17)
Grenada	0.71 (0.59, 0.88)^a^	0.1415 (1.29)	0.0413 (7.65)
Guatemala	0.77 (0.62, 0.98)^a^	1.1543 (5.15)	0.0293 (2.22)
Guyana	1.05 (0.87, 1.28)	0.3054 (6.54)	0.0146 (2.02)
Haiti	0.83 (0.63, 1.12)	0.0674 (3.40)	0.0037 (2.63)
Honduras	0.77 (0.65, 0.96)^a^	0.2871 (5.73)	0.0137 (4.66)
Jamaica	0.95 (0.79, 1.14)	0.9421 (2.58)	–-
Mexico	1.02 (0.90, 1.18)	1.6662 (9.33)	–-
Nicaragua	0.82 (0.64, 1.10)	0.3045 (4.47)	0.0087 (1.87)
Panama	0.80 (0.67, 0.98)^a^	0.8582 (4.90)	0.0330 (2.92)
Paraguay	1.03 (0.82, 1.31)	0.1429 (2.83)	0.0173 (2.37)
Peru	0.82 (0.67, 1.04)	0.8454 (6.91)	–-
Suriname	0.89 (0.74, 1.07)	1.5873 (3.01)	–-
Trinidad and Tobago	0.91 (0.76 1.11)	2.7490 (1.35)	0.4248 (2.23)
Uruguay	0.79 (0.61, 1.06)	1.7012 (8.61)	–-
Venezuela	0.70 (0.41, 1.02)	6.6656 (13.73)	–-

The values in parenthesis in columns 3 and 4 are the *t*-values associated to the estimated coefficients

*Mean evidence of mean reversion at the 95% level

Focusing next on the values of *d*, we observe that mean reversion takes place in the cases of Ecuador (*d* = 0.64), Belize (*d* = 0.68), Grenada (0.71), Guatemala (0.77), Panama (0.80), and Dominica (0.83), while in the rest of the cases, the *I*(1) hypothesis cannot be rejected. Thus, for these six countries where mean reversion is obtained, if there is a shock in the series of emissions per capita, its effect will disappear by itself in the long run though it may take some time to disappear completely.

Allowing for autocorrelated errors using the model of Bloomfield (1973) (Tables 9 and 10), the time trend is required in 17 countries, and the highest coefficients correspond to Guyana (0.1048), Barbados (*d* = 0.0602) and Chile (0.0483). Mean reversion occurs in five countries; Antigua and Barbuda (0.08), Nicaragua (0.41), Uruguay (0.43), Dominican Republic (0.44), and Haiti (0.55), all small islands, and the unit root null is rejected in favor of *d* > 1 only in the case of Mexico (1.31). In this context of autocorrelated errors, the intervals are wide and due to this, there are two countries where both the *I*(0) and the *I*(1) hypotheses cannot be rejected: Paraguay and Venezuela.

**Table 9:** Estimates of *d* under the assumption of autocorrelated errors

Country: CO_2_ per capita	No terms	An intercept	An intercept and a time trend
Antigua and Barbuda	0.27 (− 0.41, 0.76)	**0.08 (− 0.26, 0.70)**	− 0.03 (− 0.47, 0.74)
Argentina	0.74 (0.16, 1.10)	0.67 (0.35, 1.14)	**0.68 (0.28, 1.16)**
Aruba	0.70 (0.26, 1.26)	**0.81 (0.41, 1.26)**	0.79 (0.36, 1.26)
Bahamas	**1.33 (0.94, 1.92)**	1.32 (0.90, 1.90)	1.32 (0.90, 1.90)
Barbados	0.68 (0.44, 1.44)	0.91 (0.64, 1.52)	**0.90 (0.43, 1.52)**
Belize	1.02 (0.30, 1.53)	**0.76 (0.31, 1.45)**	0.88 (0.53, 1.40)
Bolivia	1.04 (0.67, 1.58)	0.98 (0.57, 1.66)	**1.00 (0.54, 1.67)**
Brazil	0.57 (0.36, 1.10)	0.87 (0.59, 1.73)	**0.75 (0.18, 1.69)**
British Virgin Islands	**0.96 (0.68, 1.38)**	1.04 (0.78, 1.44)	1.02 (0.67, 1.43)
Chile	0.83 (0.60, 1.23)	0.74 (0.53, 1.07)	**0.70 (0.43, 1.07)**
Colombia	0.89 (0.63, 1.25)	**1.07 (0.24, 1.46)**	1.07 (0.74, 1.43)
Costa Rica	0.46 (0.32, 1.09)	0.64 (0.46, 1.04)	**0.51 (0.07, 1.05)**
Cuba	0.91 (0.67, 1.31)	**0.97 (0.66, 1.41)**	0.97 (0.69, 1.41)
Dominica	**1.08 (0.86, 1.48)**	1.17 (0.92, 1.63)	1.23 (0.86, 1.63)
Dominican Republic	0.47 (0.36, 0.94)	0.62 (0.46, 0.90)	**0.44 (0.08, 0.93)**
Ecuador	0.53 (0.29, 1.11)	0.69 (0.40, 1.18)	**0.63 (0.27, 1.17)**
El Salvador	0.81 (0.60, 1.36)	0.99 (0.74, 1.42)	**0.99 (0.64, 1.41)**
Grenada	0.94 (0.80, 1.22)	1.01 (0.86, 1.27)	**1.01 (0.75, 1.30)**
Guatemala	0.91 (0.57, 1.24)	0.71 (0.08, 1.24)	**0.81 (0.32, 1.25)**
Guyana	1.22 (0.72, 1.70)	1.07 (0.40, 1.73)	**1.08 (0.41, 1.74)**
Haiti	0.66 (0.43, 1.02)	0.67 (0.46, 1.04)	**0.55 (0.24, 1.00)**
Honduras	0.81 (0.60, 1.17)	0.92 (0.73, 1.24)	**0.89 (0.60, 1.26)**
Jamaica	1.18 (0.79, 1.67)	**1.20 (0.77, 1.73)**	1.16 (0.79, 1.64)
Mexico	0.85 (0.43, 1.17)	**1.31 (1.05, 1.70)**	1.31 (1.03, 1.70)
Nicaragua	0.61 (0.04, 1.01)	0.35 (0.06, 0.78)	**0.41 (0.00, 0.82)**
Panama	1.00 (0.75, 1.34)	0.96 (0.66, 1.31)	**0.96 (0.70, 1.30)**
Paraguay	0.79 (0.41, 1.26)	0.66 (0.45, 1.21)	**0.67 (− 0.22, 1.21)**
Peru	0.96 (0.74, 1.33)	**0.83 (0.44, 1.21)**	0.84 (0.56, 1.22)
Suriname	1.14 (0.86, 1.57)	**1.13 (0.76, 1.60)**	1.13 (0.82, 1.60)
Trinidad and Tobago	**0.92 (0.65, 1.40)**	1.06 (0.77, 1.49)	1.06 (0.51, 1.60)
Uruguay	0.68 (0.36, 1.07)	**0.43 (0.10, 0.90)**	0.43 (0.10, 0.90)
Venezuela	0.41 (0.04, 0.92)	**0.28 (− 0.12, 1.10)**	0.53 (− 0.21, 1.09)

The values in parenthesis refer to the 95% confidence band of the non-rejection values of *d* using Robinson’s (1994) tests. The values in bold refer to the selected specifications in relation with the deterministic terms

**Table 10:** Estimated coefficients in the selected models in Table 9

Country: CO_2_ per capita	No terms	An intercept	An intercept and a time trend
Antigua and Barbuda	0.08 (− 0.26, 0.70)^a^	4.898 (12.73)	–-
Argentina	0.68 (0.28, 1.16)	2.448 (15.75)	0.0306 (4.32)
Aruba	0.81 (0.41, 1.26)	11.539 (3.08)	–-
Bahamas	1.32 (0.90, 1.90)	–-	–-
Barbados	0.90 (0.43, 1.52)	0.7016 (2.11)	0.0602 (2.01)
Belize	0.76 (0.31, 1.45)	0.5852 (3.91)	–-
Bolivia	1.00 (0.54, 1.67)	0.2454 (2.63)	0.0286 (2.38)
Brazil	0.75 (0.18, 1.69)	0.6237 (8.59)	0.0278 (6.91)
British Virgin Islands	0.96 (0.68, 1.38)	–-	–-
Chile	0.70 (0.43, 1.07)	1.6232 (9.54)	0.0483 (5.91)
Colombia	1.07 (0.24, 1.46)	1.0111 (11.23)	–-
Costa Rica	0.51 (0.07, 1.05)	0.3842 (4.49)	0.0229 (8.28)
Cuba	0.97 (0.66, 1.41)	1.9148 (9.64)	–-
Dominica	1.08 (0.86, 1.48)	–-	–-
Dominican Republic	0.44 (0.08, 0.93)^a^	0.2963 (2.21)	0.0376 (9.42)
Ecuador	0.63 (0.27, 1.17)	0.3471 (1.62)	0.0365 (4.21)
El Salvador	0.99 (0.64, 1.41)	0.2115 (3.73)	0.0125 (1.71)
Grenada	1.01 (0.75, 1.30)	0.2057 (1.79)	0.0393 (2.56)
Guatemala	0.81 (0.32, 1.25)	1.1451 (5.02)	0.0297 (1.96)
Guyana	1.08 (0.41, 1.74)	0.3058 (6.58)	0.1048 (1.89)
Haiti	0.55 (0.24, 1.00)^a^	0.0568 (3.20)	0.0036 (5.97)
Honduras	0.89 (0.60, 1.26)	0.2885 (5.56)	0.0138 (3.05)
Jamaica	1.20 (0.77, 1.73)	0.8110 (2.30)	–-
Mexico	1.31 (1.05, 1.70)	0.8110 (2.30)	–-
Nicaragua	0.41 (0.00, 0.82)^a^	1.6632 (10.10)	0.0076 (5.10)
Panama	0.96 (0.70, 1.30)	0.8461 (4.72)	0.0345 (1.73)
Paraguay	0.67 (− 0.22, 1.21)	0.1348 (2.68)	0.0157 (7.05)
Peru	0.83 (0.44, 1.21)	0.8417 (6.85)	–-
Suriname	1.13 (0.76, 1.60)	1.4635 (2.81)	–-
Trinidad and Tobago	0.92 (0.65, 1.40)	–-	–-
Uruguay	0.43 (0.10, 0.90)^a^	1.7609 (15.88)	–-
Venezuela	0.28 (− 0.12, 1.10)	5.9178 (30.65)	–-

The values in parenthesis in columns 3 and 4 are the t-values associated to the estimated coefficients

^a^Mean evidence of Mean reversion at the 95% level

As a robustness approach, in the final part of the manuscript, we extend the analysis by replacing the linear trend in (6) by a non-linear one of the form of the Chebyshev polynomials in time, with the disturbances still being integrated of order *d*. In particular, the model used is now

10 $$\begin{array}{cc}{y}_{t}=\sum \limits_{i=0}^{m}{\theta }_{i}{P}_{i,N}\left(t\right)+{x}_{t},& t=\text{1,2,..}.\end{array}$$

where *m* indicates the order of the Chebyshev polynomial ${P}_{i,N}\left(t\right)$ defined as:

11 $$\begin{array}{ccc}{P}_{i,N}\left(t\right)=\sqrt{2}\mathrm{cos}[i\pi (t-0.5)/N];& t=\mathrm{1,2},\dots ,N;& i=\mathrm{1,2},\end{array}\dots$$

with ${P}_{0,N}\left(t\right)=1$. If *m* = 0 in Eq. (10), then, the model contains only an intercept; if *m* = 1, it contains a linear structure, and if *m* > 1, the model becomes non-linear, where the higher the value of *m* is, the higher is the non-linear structure.^2^ The parameters ${\theta }_{i},i=\mathrm{1,2}\dots m$ are the non-linear parameters where the significance of *m* > 1 parameters implies non-linearity of the time series. Cuestas and Gil-Alana (2016) proposed a testing procedure for the integration order in a model given by Eqs. (10) and (5). See Hamming (1973) and Smyth (1998) for a detailed description of these polynomials. Bierens (1997) uses them in the context of unit root testing. The latter author proposes several unit root tests, which account for a drift and a unit root under the null hypothesis and stationarity around a linear or non-linear trend under the alternative. Hence, within the analysis of the order of integration of the variables, Bierens (1997) unit root tests allow us to test whether the process is linear or non-linear trend stationary. In addition, Bierens and Martins (2010) propose the use of Chebyshev polynomials in the framework of time-varying cointegrating parameters. There are several advantages in using these polynomials; first, since they are orthogonal, it avoids the problem of near collinearity in the regressor matrix in comparison with using regular time polynomials. Second, according to Bierens (1997) and Tomasevic et al. (2009), it is possible to approximate highly non-linear trends with a rather low degree of polynomials. Finally, given their particular shape, they are good to approximate cyclical behavior. The results for both CO_2_ emissions and CO_2_ emissions per capita are respectively reported across Tables 11 and 12.

**Table 11:** Estimated values in an *I*(*d*) model with non-linear trends. CO_2_ emissions

Country	*d*	*θ*_0_	*θ*_1_	*θ*_2_	*θ*_3_
Antigua and Barbuda	0.73 (0.46, 1.08)	0.182 (0.73)	− 0.050 (− 0.36)	0.048 (0.51)	0.063 (0.90)
Argentina	0.89 (0.63, 1.22)	**115.61 (7.10)**	** − 40.973 (− 4.79)**	2.381 (0.45)	** − 8.421 (− 2.28)**
Aruba	0.91 (0.75, 1.14)	0.737 (0.75)	− 4.312 (− 0.76)	0.115 (0.37)	0.242 (1.12)
Bahamas	0.77 (0.63, 0.97)^*^	1.816 (0.86)	0.603 (0.51)	− 0.320 (− 0.42)	** − 1.187 (− 2.11)**
Barbados	0.77 (0.58, 1.02)	**0.817 (4.87)**	** − 0.379 (− 4.02)**	− 0.052 (− 0.87)	− 0.017 (− 1.52)
Barbados	0.49 (0.25, 0.78)^*^	**0.301 (11.21)**	** − 0.170 (− 10.55)**	0.014 (1.09)	− 0.016 (− 1.52)
Bolivia	0.81 (0.64, 1.02)	**0.384 (4.71)**	** − 5.877 (− 5.85)**	**1.788 (2.88)**	** − 1.120 (− 2.49)**
Brazil	1.02 (0.75, 1.32)	**241.80 (4.33)**	** − 133.05 (− 4.02)**	17.754 (1.10)	** − 22.65 (− 2.11)**
British Virgin Islands	0.89 (0.67, 1.14)	**0.084 (3.91)**	** − 0.068 (− 5.52)**	0.009 (1.38)	0.002 (0.37)
Chile	1.00 (0.73, 1.39)	**35.807 (3.22)**	** − 21.837 (− 3.34)**	**7.086 (2.16)**	− 1.040 (− 0.47)
Colombia	0.72 (0.56, 0.93)^*^	**53.860 (3.22)**	** − 20.928 (− 6.14)**	0.750 (0.32)	** − 5.993 (− 3.44)**
Costa Rica	0.87 (0.63, 1.20)	**3.783 (4.90)**	** − 2.584 (− 5.87)**	0.375 (1.47)	− 0.107 (− 0.59)
Cuba	0.82 (0.53, 1.15)	**26.348 (5.83)**	− 2.448 (− 0.95)	** − 3.451 (− 2.21)**	** − 3.311 (− 2.94)**
Dominica	0.51 (0.32, 0.74)^*^	**0.075 (11.03)**	** − 0.054 (− 14.59)**	**0.014 (4.72)**	− 0.003 (− 1.42)
Dominican Republic	0.99 (0.73, 1.32)	**11.327 (2.92)**	** − 7.805 (− 3.42)**	0.574 (0.49)	− 0.043 (− 0.05)
Ecuador	0.28 (0.08, 0.54)^*^	**19.213 (23.43)**	** − 12.046 (− 20.44)**	**0.975 (1.86)**	** − 2.463 (− 5.26)**
El Salvador	0.85 (0.69, 1.07)	**3.123 (4.36)**	** − 2.130 (− 5.26)**	0.163 (0.68)	0.230 (1.34)
Grenada	0.23 (0.04, 0.48)^*^	**0.129 (41.77)**	** − 0.089 (− 38.32)**	**0.018 (8.41)**	0.001 (0.51)
Guatemala	1.25 (1.11, 1.40)	3.928 (0.75)	− 2.468 (− 0.75)	0.855 (0.68)	− 0.240 (− 0.32)
Guyana	0.75 (0.58, 0.97)^*^	**1.387 (4.43)**	** − 0.321 (− 1.82)**	0.039 (0.33)	** − 0.200 (− 2.32)**
Haiti	0.81 (0.52, 1.17)	**1.330 (3.77)**	** − 0.824 (− 4.14)**	**0.285 (2.31)**	** − 0.207 (− 2.32)**
Honduras	0.34 (0.13, 0.65)^*^	**4.078 (30.58)**	** − 3.015 (− 33.44)**	**1.045 (13.37)**	** − 0.390 (− 5.61)**
Jamaica	0.93 (0.78, 1.13)	**5.317 (1.97)**	− 1.881 (− 1.20)	− 0.810 (− 0.95)	0.027 (0.04)
Mexico	0.76 (0.56, 0.99)^*^	**300.39 (11.67)**	** − 137.56 (− 9.52)**	** − 20.98 (− 2.23)**	− 10.05 (− 1.44)
Nicaragua	0.76 (0.54, 1.08)	**2.606 (5.61)**	** − 1.416 (− 5.43)**	0.227 (1.34)	** − 0.240 (− 1.91)**
Panama	0.49 (0.27, 0.76)^*^	**4.879 (13.87)**	** − 2.923 (− 13.84)**	**1.101 (6.49)**	** − 0.833 (− 5.86)**
Paraguay	1.28 (1.11, 1.54)	1.761 (0.66)	− 1.174 (− 0.69)	0.218 (0.35)	− 0.087 (− 0.23)
Peru	0.37 (0.01, 0.77)^*^	**27.170 (18.29)**	** − 11.478 (− 11.75)**	**3.391 (4.06)**	** − 4.433 (− 6.03)**
Suriname	0.75 (0.56, 0.99)^*^	**1.645 (4.27)**	** − 0.417 (− 1.93)**	− 0.120 (− 0.84)	** − 0.290 (− 2.74)**
Trinidad and Tobago	0.90 (0.74, 1.09)	**20.802 (3.22)**	** − 12.647 (− 3.41)**	2.236 (1.07)	** − 2.381 (− 1.64)**
Uruguay	0.71 (0.48, 1.00)	**4.940 (4.78)**	− 0.582 (− 1.00)	0.465 (1.18)	− 0.259 (− 0.86)
Venezuela	0.73 (0.44, 1.05)	**108.61 (5.61)**	** − 40.168 (− 3.70)**	− 4.262 (− 0.58)	7.896 (1.44)

**Table 12:** Estimated values in an *I*(*d*) model with non-linear trends. CO_2_ emissions per capita

Country	*D*	*θ*_0_	*θ*_1_	*θ*_2_	*θ*_3_
Antigua and Barbuda	0.73 (0.46, 1.09)	2.243 (0.57)	0.027 (0.01)	0.309 (0.21)	− 0.849 (− 0.76)
Argentina	0.97 (0.75, 1.26)	**3.402 (5.38)**	− 0.453 (− 1.22)	− 0.038 (− 0.20)	** − 0.228 (− 1.76)**
Aruba	0.75 (0.59, 0.97)^*^	10.668 (1.51)	− 2.184 (− 0.55)	0.726 (0.28)	2.366 (1.22)
Bahamas	0.79 (0.64, 0.98)^*^	6.613 (0.61)	5.5054 (0.83)	− 1.127 (− 0.29)	** − 5.557 (− 1.99)**
Barbados	0.77 (0.57, 1.01)	**3.041 (4.82)**	** − 1.259 (− 3.55)**	− 0.267 (− 1.17)	− 0.062 (− 0.37)
Belize	0.58 (0.37, 0.82)^*^	**1.288 (7.97)**	** − 0.296 (− 3.18)**	** − 0.170 (− 2.42)**	− 0.040 (− 0.72)
Bolivia	0.81 (0.64, 1.03)	**0.956 (4.66)**	** − 0.452 (− 3.90)**	0.072 (1.01)	** − 0.097 (− 1.84)**
Brazil	1.12 (0.87, 1.40)	**1.523 (3.37)**	** − 0.490 (− 1.78)**	0.014 (0.12)	** − 0.143 (− 1.88)**
British Virgin Islands	0.76 (0.58, 0.99)^*^	**3.197 (2.03)**	** − 1.593 (− 1.80)**	− 0.855 (− 1.48)	0.602 (1.41)
Chile	1.09 (0.83, 1.47)	**1.289 (2.21)**	− 0.858 (− 1.37)	0.372 (1.32)	0.024 (0.13)
Colombia	0.66 (0.48, 0.89)^*^	**1.549 (13.42)**	** − 0.158 (− 2.43)**	0.053 (− 1.16)	** − 0.138 (− 3.85)**
Costa Rica	0.98 (0.76, 1.29)	**0.995 (2.85)**	** − 0.390 (− 1.91)**	− 0.010 (− 0.10)	− 0.041 (− 0.59)
Cuba	0.83 (0.55, 1.14)	**2.667 (5.97)**	0.013 (0.05)	** − 0.272 (− 1.78)**	** − 0.309 (− 2.81)**
Dominica	0.52 (0.33, 0.75)^*^	**1.054 (11.43)**	** − 0.765 (− 14.1)**	**0.200 (4.67)**	− 0.034 (− 0.97)
Dominican Republic	0.97 (0.73, 1.29)	**1.365 (2.90)**	** − 0.662 (− 2.41)**	− 0.076 (− 0.54)	− 0.003 (− 0.03)
Ecuador	0.37 (0.14, 0.66)^*^	**1.624 (15.29)**	** − 0.596 (− 8.54)**	** − 0.173 (− 2.90)**	** − 0.206 (− 3.93)**
El Salvador	0.86 (0.70, 1.07)	**0.549 (3.82)**	** − 0.283 (− 3.46)**	0.019 (0.39)	0.041 (1.20)
Grenada	0.32 (0.15, 0.56)^*^	**1.245 (29.69)**	** − 0.819 (− 28.4)**	**0.126 (4.98)**	0.024 (1.08)
Guatemala	1.08 (0.89, 1.29)	**0.545 (2.12)**	− 0.178 (− 1.15)	0.031 (0.44)	− 0.013 (− 0.29)
Guyana	0.72 (0.55, 0.95)^*^	**1.898 (5.05)**	** − 0.363 (− 1.72)**	0.091 (0.64)	** − 0.211 (− 1.96)**
Haiti	0.72 (0.42, 1.07)	**0.160 (5.04)**	** − 0.060 (− 3.35)**	0.015 (1.31)	** − 0.019 (− 2.15)**
Honduras	0.67 (0.50, 0.88)^*^	**0.625 (9.62)**	** − 0.244 (− 6.27)**	**0.063 (2.30)**	− 0.033 (− 1.54)
Jamaica	0.93 (0.78, 1.13)	**2.017 (1.67)**	− 0.350 (− 0.50)	− 0.349 (− 0.92)	− 0.058 (− 0.22)
Mexico	0.81 (0.62, 1.03)	**3.438 (9.32)**	** − 0.678 (− 3.26)**	** − 0.453 (− 3.52)**	− 0.135 (− 1.45)
Nicaragua	0.79 (0.59, 1.08)	**0.545 (3.82)**	− 0.115 (− 1.43)	0.010 (0.20)	− 0.055 (− 1.50)
Panama	0.64 (0.47, 0.87)^*^	**1.654 (7.73)**	** − 0.458 (− 3.79)**	**0.187 (2.14)**	** − 0.223 (− 3.25)**
Paraguay	1.08 (0.88, 1.32)	**0.511 (1.83)**	− 0.229 (− 1.36)	0.0004 (0.01)	− 0.021 (− 0.43)
Peru	0.56 (0.31, 0.86)^*^	**1.176 (10.47)**	** − 0.131 (− 2.02)**	**0.098 (1.96)**	** − 0.174 (− 4.30)**
Suriname	0.72 (0.53, 0.96)^*^	**3.825 (4.62)**	− 0.241 (− 0.52)	** − 0.585 (− 1.89)**	** − 0.731 (− 3.08)**
Trinidad and Tobago	0.83 (0.67, 1.02)	**16.378 (3.51)**	** − 8.451 (− 3.20)**	1.125 (0.70)	** − 1.892 (− 1.65)**
Uruguay	0.72 (0.50, 1.02)	**1.621 (4.83)**	− 0.022 (− 0.11)	0.162 (1.28)	− 0.076 (− 0.80)
Venezuela	0.50 (0.13, 0.93)^*^	**5.904 (15.12)**	0.029 (0.12)	− 0.046 (− 0.25)	**0.496 (3.19)**

(*) Indicates statistical evidence of mean reversion at the 95% level

Starting with the results in Table 11 (CO_2_ emissions), we observe that non-linear structures are visible in a number of cases. In particular, there are 12 countries with at least one of the two non-linear coefficients (*θ*_2_ and *θ*_3_) being statistically significant; for a group of 7 countries, the two coefficients are significant, and for the remaining 13 countries, there is no evidence of non-linearities. On the other hand, the orders of integration are smaller than in the previous tables, and mean reversion takes place in 12 countries (Bahamas, Belize, Colombia, Dominica, Ecuador, Grenada, Guyana, Honduras, Mexico, Panama, Peru, and Suriname). Of these twelve countries, all except two display a non-linear pattern.

Table 12 reports the results for the ${\mathrm{CO}}_{2}$ emissions per capita. Again, non-linearities are observed in 19 countries, and in five of them (Cuba, Ecuador, Panama, Peru, and Suriname) with the two non-linear coefficients being statistically significant. Similarly to the previous table, of the fourteen countries where mean reversion occurs, in twelve of them, non-linearities are detected, implying a clear relation between the two issues.

As a robustness method, we use alternative non-linear *I*(*d*) approaches like the one based on Fourier functions in time (Gil-Alana and Yaya 2021) and another one that employs neural networks (Yaya et al. 2021), and, though the results differ quantitatively in some cases, the conclusions were very similar to those reported across Tables 11 and 12.

## Concluding comments and recommendations

In this article, we have examined ${\mathrm{CO}}_{2}$ emissions in Latin America and the Caribbean countries using a long memory model based on fractional integration. This technique is appropriate first if we want to determine the nature of the shocks, which are transitory if the differencing parameter is smaller than 1. In addition, not taking into account this long memory feature (i.e., a positive order of integration) of the data may produce spurious results in the time trend coefficients of the models.

We can summarize the main results reported in this work as follows: Starting with the ${\mathrm{CO}}_{2}$ emissions, and focusing first on the case of white noise errors, Belize along with Ecuador, Granada, Colombia, Peru, and Dominica display mean reversion and thus transitory shocks. It is worth noting that for all these countries, we also observe significant positive trends, being particularly important in the cases of Colombia, Peru, and Ecuador that present the highest coefficients. Noting that the differencing parameter is smaller than 1 in these cases, it is expected that in the event of shocks, the series will reverse to their original trends. In the rests of the cases (and particularly Guatemala and Paraguay where the differencing parameter is significantly higher than 1), the effects of shocks are expected to be permanent, which may alter the trend of the variable. Still in this context of white noise errors, the time trend coefficient is found to be significantly positive in 24 out of the 32 countries examined, with the highest values obtained for Brazil (6.707), Mexico (6.398), and Argentina (2.213), which are three of the most industrialized and populated countries in Latin America and the Caribbean. The eight countries where the time trends are insignificant are Antigua and Barbuda, Aruba, Bahamas, Cuba, Jamaica, Paraguay, Uruguay, and Venezuela.

Allowing the error term to be autocorrelated, and thus, including more structure in the model, evidence of reversion to the mean is found in the cases of Antigua and Barbuda, Belize, Uruguay, Dominican Republic, and Nicaragua, and in all of these except Antigua and Barbuda, the time trend coefficient is significantly positive. For the rest of the countries, we reject this hypothesis (mean reversion) in favor of *d* being equal to or higher than 1. The time trends are significant in 17 countries, and the highest coefficients correspond to Brazil and Argentina. Performing non-linear trends of the form of the Chebyshev polynomials in time, we note that mean reversion is found in a large number of cases.

This implies a complex scenario in terms of intervention. Likewise, the cases of countries such as Brazil, Argentina, Colombia, Chile, and Venezuela show a statistically significant trend over time. This may be related to the growth of hydrocarbon and mining sectors that represent high percentages in the net exports of the mentioned countries.

Considering that in spite of facing important challenges on climate change and not being able to rule out permanent effects in their emissions and effects caused by ${\mathrm{CO}}_{2}$ emissions, countries such as Antigua and Barbuda, Aruba, Bahama, British Virgin Islands, Cuba, Dominica, Guatemala, Jamaica, Mexico, Suriname, and Trinidad and Tobago do not present a clear trend regarding future behavior. Although as detailed in the WMO (2021), sea level rise poses a great risk to low-lying coastal areas in the Latin American and Caribbean region, and people living in these areas are particularly at risk. This risk may increase due to a possible doubling of the frequency of even small rises in water level (0.1 to 0.2 m).

Now, taking into account the values of per capita ${\mathrm{CO}}_{2}$ emissions, and starting once more with the model with no autocorrelation for the error term, mean reversion takes place at Belize, Dominica, Ecuador, Grenada, Guatemala, Honduras, and Panama, all small Central American countries; lack of this property is observed in all the remaining countries. Focusing on the time trend coefficient, this is significant in 19 countries, and the most significant coefficients are now the British Virgin Islands, Barbados, and Trinidad and Tobago, confirming that particularly these small Caribbean states show the highest trend levels that might have pronounced impact in climatic terms. This also implies the need for the effective application of efficient projects in terms of emission regulations, since they can become permanent effects for the ecosystem.

If autocorrelation is permitted, Antigua and Barbuda, Dominican Republic, Haiti, Nicaragua, and Uruguay are the only countries showing reversion to the mean. Thus, permanent effects of shocks are expected in the majority of the countries. The time trends are now significant in 17 countries, the highest coefficients being observed in Guyana and Barbados.

As a general conclusion, we observe very few countries displaying mean reversion (see Table 13 for a summary of the results). We see that the results are very similar to the two variables (${\mathrm{CO}}_{2}$ emissions and emissions per capita), and though there are some differences in the countries depending on the modelization of the error term, most of these are relatively small countries in Central America and the Caribbean. For the rest of the countries, shocks are expected to be permanent. Thus, in the event of negative exogenous shocks (increasing the number of emissions), strong policy measures will be required to recover the series to their original levels. On the other hand, if the shock is positive, reducing the emissions, there is no need for strong actions on the part of the authorities since the series will remain at the lower established level.

**Table 13:** Summary results in terms of persistence: countries showing reversion to the mean

${\mathrm{CO}}_{2}$ emissions	
No autocorrelation	With autocorrelation
Belice (0.47)	Antigua & Barbuda (0.20)
Ecuador (0.48)	Belize (0.29)
Grenada (0.72)	Dominican Republic (0.42)
Colombia (0.76)	Nicaragua (0.48)
Peru (0.76)	Uruguay (0.39)
Dominica (0.76)	
${\mathrm{CO}}_{2}$ emissions per capita	
No autocorrelation	With autocorrelation
Ecuador (0.64)	Antigua and Barbuda (0.08)
Belice (0.68)	Nicaragua (0.41)
Grenada (0.71)	Uruguay (0.43)
Guatemala (0.77)	Dominican Republic (0.44)
Honduras (0.77)	Haiti (0.55)
Panama (0.80)	
Dominica (0.83)	

In general terms, we have to highlight that efforts have to be taken to remove the positive time trends observed in some countries along with the high levels of persistence observed in many countries that indicate the permanent nature of the shocks in the series of ${\mathrm{CO}}_{2}$ emissions. It is clear that in Latin America, although there are countries with high levels of development and therefore higher levels of carbon dioxide emissions, the region as a whole is receiving direct and indirect impacts of climate change as detailed in WMO (2021) and confirmed by this research.

It can be seen that in the particular case of Latin America and the Caribbean, it has become one of the regions of the world where climate shocks are expected to be increasingly intense in the form of heat waves, reduced crop yields, forest fires, destruction of coral reefs, and phenomena related to extreme sea levels.

In conclusion, and taking into consideration the socio-environmental conditions of the region, climate change must be considered as one of the main threats to Latin American societies, as it may cause structural alterations in agriculture and food systems, considering the projected reductions in yields of most crops. In addition, as mentioned by WMO (2021), the projected historical impacts of climate change can be related from a sectoral perspective that includes direct impacts on the agricultural sector and the availability of water resources; impacts on forest and ecosystem services; direct impacts on socioeconomic development, infrastructure, and forced displacement; and loss of living conditions, limiting the development conditions of populations with high levels of inequality and poverty, which is a common denominator in the Latin American and Caribbean region. It is therefore urgent to adopt effective mitigation and emission reduction measures and policies that include risk prevention and management measures, particularly in countries with permanent changes in their structures and to reflect on how these decisions do not indirectly affect the small states of the region, which have historically played a marginal role in international decision-making.

## Data Availability

The datasets generated during and/or analyzed during the current study are available from the corresponding author on reasonable request.

## Footnotes

1 1We are clearly referring to shocks that increase the number of emissions. The contrary happens if the shock is negative: actions should then be taken if the series is stationary since the shock will otherwise disappear by itself in the long run.

2 2A detailed description of the Chebyshev polynomails in time can be found in Hamming (1973) and Smyth (1998). Bierens (2001) used these polynomials in the context of unit root models, and Bierens and Martins (2010) proposed its use in time-varying cointegrating models.
